# Histone deacetylase 2 knockout suppresses immune escape of triple-negative breast cancer cells via downregulating PD-L1 expression

**DOI:** 10.1038/s41419-021-04047-2

**Published:** 2021-08-07

**Authors:** Pengfei Xu, Wei Xiong, Yun Lin, Liping Fan, Hongchao Pan, Yaochen Li

**Affiliations:** 1 The Central Laboratory, Cancer Hospital of Shantou University Medical College, 7 Raoping Road, Shantou, 515041 China; 2 Guangdong Provincial Key Laboratory of Breast Cancer Diagnosis and Treatment, 7 Raoping Road, Shantou, 515041 China

**Keywords:** Breast cancer, Immune evasion

## Abstract

The PD-L1 overexpression is an important event of immune escape and metastasis in triple-negative breast cancer (TNBC), but the molecular mechanism remains to be determined. Interferon gamma (IFNγ) represents a major driving force behind PD-L1 expression in tumor microenvironment, and histone deacetylase 2 (HDAC2) is required for IFN signaling. Here, we investigated the regulation of HDAC2 on the IFNγ-induced PD-L1 expression in TNBC cells. We found the HDAC2 and PD-L1 expression in TNBC was significantly higher than that in non-TNBC, and HDAC2 was positively correlated with PD-L1 expression. HDAC2 promoted PD-L1 induction by upregulating the phosphorylation of JAK1, JAK2, and STAT1, as well as the translocation of STAT1 to the nucleus and the recruitment of STAT1 to the PD-L1 promoter. Meanwhile, HDAC2 was recruited to the PD-L1 promoter by STAT1, and HDAC2 knockout compromised IFNγ-induced upregulation of H3K27, H3K9 acetylation, and the BRD4 recruitment in PD-L1 promoter. In addition, significant inhibition of proliferation, colony formation, migration, and cell cycle of TNBC cells were observed following knockout of HDAC2 in vitro. Furthermore, HDAC2 knockout reduced IFNγ-induced PD-L1 expression, lymphocyte infiltration, and retarded tumor growth and metastasis in the breast cancer mouse models. This study may provide evidence that HDAC2 promotes IFNγ-induced PD-L1 expression, suggesting a way for enhanced antitumor immunity when targeting the HDAC2 in TNBC.

## Introduction

Triple-negative breast cancer (TNBC), accounting for 15–20% of breast cancer cases, represents a more biologically aggressive cluster with rapid proliferation, high rates of relapse, frequently occurring metastasis, and poor prognosis [1]. Unfortunately, TNBC does not respond to hormonal or HER2-targeted therapies due to the lack of molecular targeted receptors like ER, PR, and HER2/neu [2]. Hence, the clinical need of effective therapeutic approaches for TNBC patients is dramatically emerging. Indeed, immunologic escape intently engages in the progression of TNBC [3]. Notably, the expression of programmed cell death ligand 1 (PD-L1) on the surface of cancer cells, a key immune checkpoint molecule, interacts with its receptor-programmed cell death (PD-1) on immune cells, and counteracts the TCR cascade through phosphorylation of PTPN11 to neutralize cytotoxic T-cell activity [4]. Therefore, disrupting of PD-1/PD-L1 interactions by using antibodies can prevent T-cell suppression and enhance antitumor immunity both in in vitro and in vivo experiments [5]. Independent of its immunosuppressive properties, PD-L1 has recently been shown to also exert a cancer cell-intrinsic function promoting tumorigenesis, i.e., cell growth, pathogenesis, and autophagy [6, 7]. A study has shown that the PD-L1 expression is significantly higher in TNBC and HER2+ subtypes, which positively correlates with the 3^rd^ histology level and lymph node metastasis, indicating that PD-L1 is a biomarker of poor prognosis [8]. Experiments in vitro showed that the inhibition of proliferation and migration, high rate of apoptosis were observed after PD-L1 knockdown in TNBC cells [9]. To date, the clinical trials of immunotherapies based on the PD-1/PD-L1 antagonists have shown a notable and durable response in TNBC patients [10, 11].

The molecular mechanism driving PD-L1 overexpression of TNBC remains to be determined. Some cytokines, secreted by APCs and T cells, such as IFNγ, TNFα, and IL-6, as well as the PTEN/PI3K pathway, are all reported to be involved in this process [12, 13]. In particular, of them, IFNγ is the strongest inducer to elevate PD-L1 expression in tumor microenvironment, known as “adaptive immune resistance” [14, 15]. In a reciprocal way, the resistance to PD-L1 therapy is also related to the defect of IFNγ signaling pathway in tumor cells [16]. Besides, researchers analyzed the gene expression data of invasive breast cancer tissues and proved that the JAK/STAT1 pathway activated by IFNγ was positively correlated with PD-L1 expression [17]. Also, other investigators have demonstrated that PD-L1 expression was mediated through the expression and activation of both JAK2 and STAT1 [18–20]. These observations suggest that the upregulation of PD-L1 induced by IFNγ–JAK/STAT1 pathway appears to play a critical role in the immune escape of breast cancer.

As the most important epigenetic factors, histone deacetylases (HDACs) tightly controlled cancer initiation and progression by modulation of gene expression and cellular signals [21]. The inhibitors of histone deacetylases (HDACi) are already used in treatment of cancers over the past years [22]. In addition to their effects on cancer signaling, HDACi engage the host immune system and upregulate or downregulate PD-L1 expression in different types of cancer cells [23, 24], which may be related to the nonspecific inhibitory effect of pan-HDACi and the diversity of HDAC enzyme in different tumors. In this context, several studies have demonstrated that single HADC modulate PD-L1 expression. For instance, HDAC6 recruited and activated STAT3, enabling an upregulation of PD-L1, and the treatment of HDAC6 specific-inhibitors leads to retard tumor growth and downmodulate PD-L1 expression in vivo [25]. Moreover, the transcriptional complex composed with HDAC8, HOXA5, and STAT3 controls the transcriptional activation of PD-L1, and the inhibition of HDAC8 can upregulate PD-L1 expression by increasing its activity in melanoma cells [26]. Of our interest, HDAC2, the major class I enzyme, has been shown to be required for both type I and type II IFN signaling [27–29], suggesting that it may be involved in the regulation of PD-L1 expression induced by IFNγ. Meanwhile, recent study has shown that only HDAC2, but not other HDACs or SIRTs, can bind and deacetylate PD-L1 in TNBC cells, indicating the unique effect of HDAC2 in the regulation of PD-L1 function [30]. Moreover, HDAC2 has been characterized as a critical regulator in tumorigenesis, cell cycle progression and immune escape of cancer cells [31]. Additionally, previous studies reported that higher HDAC2 expression was correlated with metastasis, aggressiveness, and poor prognosis in breast cancer [32]. Unfortunately, its potential role in TNBC as immune modulator and the mechanism promoting PD-L1 induction has not been investigated. Therefore, in the present study, we aimed to explore the role of HDAC2 in PD-L1 expression induced by IFNγ in TNBC cells, which suggested a new promising target for immunotherapy in TNBC.

## Materials and methods

### Bioinformatic analysis

The expression of HDAC2 and PD-L1, as well as the correlation of these two genes in breast cancer, was evaluated using bc-GenExMiner 4.5 web server. The HDAC2 and CD274 expression in normal and breast cancer tissues, and the relationship between the expression of HDAC2 and the prognosis of breast cancer patients, was analyzed by the online tool GEPIA2.

### Cell line culture, antibodies, and reagents

The human (MDA-MB-231, BT-549, SKBR3, and T47D) and mouse (4T-1, 4T-1-luc) breast cancer cell lines were purchased from the American Type Culture Collection (ATCC) and maintained in our laboratory. All cells were routinely tested for the absence of mycoplasma using a Mycoplasma Detection Kit (Bitool, China). All cells were cultured in complete medium as our previously described method [33]. We constructed a breast cancer cell line (4T-1-luc) that stably knocks out HDAC2 (HDAC2-KO) and nontargeted control (WT) cell lines using CRISPR/Cas9 technology, as we described previously [27]. Briefly, 4T-1 cells were transfected by guide RNA (target sequences: TGAGTCATCCGGATTCTATGAGG)-encoding plasmids for two days. Multiple monoclonal cells were screened with G418 for 14 days, then the expression of HDAC2 was examined by western blotting and RT-PCR, and two successful knockout clones were selected and cultured for the further experiments. Details on the antibodies and reagents were described in the Supplementary Materials.

### RNA isolation and RT-PCR analysis

RNA isolation and cDNA synthesis were carried out using TRIzol reagent (Invitrogen, Carlsbad, CA, USA) and PrimeScript RT kit (Takara, Shiga, Japan), respectively. Quantitative PCR was performed using the primer pairs listed in Supplementary Table 1 and TB Green Premix Ex Taq II (Takara) on a CFX Connect Real-Time System (Bio-Rad, USA). The 2^−ΔΔCt^ method was used to calculate gene expression levels.

### Plasmids and siRNA transfection

The Flag-tagged human and mouse HDAC2 expression plasmids (pcDNA3.1), the small interference RNA (siRNA) targeting human HDAC2, and nontargeted control sequences were obtained from the Synbio Technologies (Suzhou, China). The siRNA primers are presented in Supplementary Table 2. Cells were cultured overnight, and then 2 μg of plasmids or 100 nM siRNA, lipofectamine 3000 (Invitrogen), and Opti-MEM medium (Invitrogen) were used to transfect into cells for 6 h. Cells were used for further experiments after culturing for 1–3 days.

### Western blotting and co-immunoprecipitation (Co-IP)

Total protein extraction and western blotting was performed using standard techniques, as our previously described method [27]. The information of primary and secondary antibodies used in this study was listed in Supplementary Materials. Co-IP Kit (#26149, Thermo Fisher Scientific, MA, USA) was used to detect the interaction of proteins according to standard procedures.

### Immunofluorescence assay

4T-1 cells growing on coverslips overnight were treated with IFNγ for the indicated time. The cells were fixed, permeabilized, and blocked with normal goat serum. After culturing with diluted antibodies overnight, cells were then incubated with Alexa Fluor 488 or Alexa Fluor 594-conjugated antibody for one hour, followed by washing in PBS and staining with DAPI. Analysis was conducted under a fluorescence microscope (Zeiss, Germany).

### Chromatin immunoprecipitation (ChIP)

The ChIP assays were conducted according to the manufacturer’s guidelines of a ChIP Assay Kit (Cat. No. P2078, Beyotime, Shanghai, China). Briefly, after fixation with formaldehyde for ten minutes to crosslink DNA and protein, 4T-1 cells were sonicated to obtain DNA fragments with the length of about 500 bp yield. The prepared chromatin was precipitated using polyclonal antibodies and normal rabbit IgG antibody overnight. The next day, the complexes were thoroughly washed, eluted, and purified. To detect the percent of enrichment, qPCR was conducted using the primers listed in Supplementary Table 3. The data were analyzed using the following method:

$${{{\mathrm{Percent}}}}\,{{{\mathrm{Input}}}} = 2\% \,\times 2^{({{{\mathrm{C}}}}[2\% \,{{{\mathrm{Input}}}}\,{{{\mathrm{Sample}}}}-{{{\mathrm{C}}}}[{{{\mathrm{T}}}}]{{{\mathrm{IP}}}}\,{{{\mathrm{Sample}}}}])}$$

C [T] = threshold cycle of PCR

### Cell proliferation and colony formation

4T-1 cells (500 cells/well) were plated in 96-well plates. Then CCK-8 reagent (GK10001, GlpBio, America) was added and cultured for 1–4 h. At the indicated time points, the absorbance was read at 450 nm using an automatic plate reader. Cells (1000 cells/well) were cultured on six-well plates for two weeks to assess the clone-forming capacity.

### Wound healing

Cells were grown to about 90% confluence before the scratch wound was made by a 2 mm-wide tip. The photographs of each group were taken (×40) at the indicated times. The injury lines were measured by an average of five random widths.

### Transwell assay

Transwell methods and culture inserts (8 μM pore size, BD Biosciences) were used to analyze the cell migration. Cells (5 × 10^4^/well) were plated into the upper chamber in serum-free medium, and the bottom chamber containing complete medium. After culturing for 24 h, cells were stained with 0.1% crystal violet, and the positive cells from five fields were counted and analyzed under a microscope.

### Cell cycle analysis

Cells were harvested, washed, and fixed in 70% ethanol at 4°C overnight. After staining with PI and RNase A for thirty minutes, then cells and data were analyzed by flow cytometry (BD Biosciences, USA) and FlowJo software (Tree Star), respectively.

### Tumor models

BALB/c and BALB/c-nu mice (female, 6–8 weeks old) obtained from Beijing Vital River Laboratories were bred in SPF facilities. 4T-1-luc cells (3 × 10^6^/mouse) were intravenously injected into mice to construct a lung metastasis model (5–10 mice per group). One hundred μL of cell suspension (3 × 10^6^/mouse) were implanted into the 4th mammary pad of mice for tumorigenicity. Mice received intraperitoneal luciferin (10 mg/kg) injection and imaged at various time points using a bioluminescent imaging system (PerkinElmer, USA). Living Image Software (PerkinElmer) was used to analyze the obtained images. The size of tumor was assessed every 3–4 days, using the formula width^2^ × length × 0.5. For the lung metastasis and tumorigenicity assay, mice were euthanized during days 13 and 33 post injection, respectively. The lung and tumor tissues were dissected, photographed, weighed, isolated, sectioned, and stained. The in vivo studies were approved by the Medical Ethics Committee of Shantou University.

### Immunohistochemistry (IHC)

IHC analysis was conducted according to standard procedures. Briefly, the sections of tissue blocks were deparaffinized, rehydrated, washed, and subjected to citrate buffer. After blocking with 3% hydrogen peroxide, the sections were stained with the diluted primary antibodies, followed by incubation with the HRP-conjugated secondary antibodies and DAB complex.

### Flow cytometry

Cells were collected, washed and resuspended in PBS with CD274-PE or isotype-control antibodies for half an hour, then the mean fluorescence intensity (MFI) of cells (50,000 events/sample) were measured by flow cytometry (BD Biosciences, USA) to detect the PD-L1 expression. To analyze the infiltrating lymphocytes of tumors, the tumor tissues from mouse models were cut and then digested for one hour, as our previously described method [27]. After preparing the single-cell suspension by filteration through a 70-μm strainer (BD), cells of each group were washed and resuspended with PBS. After blocking with α-CD16/32 antibody for 10 min, cells were stained and incubated with antibodies (Supplemental Materials) for half an hour, and then washed in PBS twice. At least 100,000 events were collected using Cytek Aurora (Cytek Biosciences, USA) and subsequently analyzed using FlowJo software. Gating strategy: the dead cells and debris were excluded based on cell size, the cells were first gated as CD45 populations, then CD45+CD3+ cells, and finally gated as CD3+CD4+, CD3+CD4+FOXP3+, CD3+CD8+, CD3+CD8+CD69+, and CD3+CD8+CD107+ cells, respectively.

### Statistical analysis

All values were presented as mean ± SD as experiments were at least independently carried out in triplicate or biological replicates. Data were analyzed using Student’s *t*-test (GraphPad Prism 7.0 Software, San Diego, CA, USA). The *P* < 0.05 was considered statistically significant. Statistical significance was defined as ns, no significant difference; *P < 0.05; **P < 0.01; ***P < 0.001; ****p < 0.0001.

## Results

### The expression of HDAC2 and PD-L1 was higher in TNBC than that in other breast cancer subtypes

**Fig. 1:**
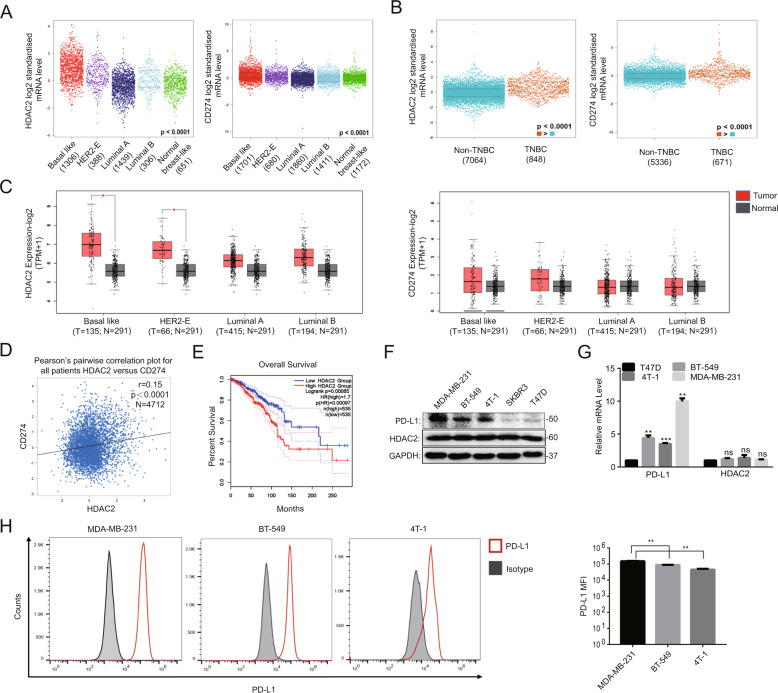
The HDAC2 and PD-L1 expression was higher in TNBC than that in other breast cancer subtypes. **A**, **B** The bc-GenExMiner v 4.5 analysis for HDAC2 and PD-L1 expression in different breast cancer subtypes (**A**), as well as in TNBC and non-TNBC subtypes (**B**). **C** The HDAC2 and PD-L1 expression between normal and breast cancer tissues was determined by GEPIA2. **D** The correlation between HDAC2 and PD-L1 expression was analyzed by bc-GenExMiner v 4.5. **E** The relationship between HDAC2 expression and the prognosis of breast cancer patients was determined by GEPIA2. **F**, **G** Western blotting (**F**) and RT-PCR (**G**, *n* = 3) detected the HDAC2 and PD-L1 expression in the cells of breast cancer. **H** The flow cytometry results showed the cell surface expression of PD-L1 in TNBC cells (*n* = 3).

In initial experiments, we elucidated the expression of HDAC2 and PD-L1 (CD274) from two online websites, including bc-GenExMiner v4.5 and GEPIA2. As shown in Fig. 1A, the expression of both of those two genes was the highest in basal-like subtypes than that in other subtypes. We then compared the TNBC and non-TNBC group, and found that the expression of those two genes in TNBC was significantly higher than that in non-TNBC (Fig. 1B). Meanwhile, we also used the online tool GEPIA2 to analyze the HDAC2 and PD-L1 expression in breast cancer and normal breast tissues. The results showed that the expression of those two genes in basal-like and HER-2 subtypes was relatively higher than that in normal tissues, but the differences of PD-L1 between tumor and normal tissues was not statistically significant (Fig. 1C). In addition, correlation analysis from bc-GenExMiner v4.5 revealed that the expression of HDAC2, as well as JAK1, JAK2, and STAT1, was significantly correlated with that of PD-L1 in breast cancer (Fig. 1D, Fig. S1). Moreover, the survival analysis from GEPIA2 showed that high expression of HDAC2 was associated with poor prognosis of breast cancer patients (Fig. 1E).

Next, western blotting and RT-PCR were carried out to test HDAC2 and PD-L1 expression in various breast cancer cells, including 3 TNBC cells (MDA-MB-231, BT-549, and 4T-1) and 2 non-TNBC cells (SKBR3, T47D). As shown in Fig. 1F and G, the protein and mRNA expression of PD-L1 was higher in TNBC cells than that in non-TNBC cells, especially in MDA-MB-231 cells. Consistent with the above results, TNBC cells express higher levels of PD-L1 than that of non-TNBC cells, while HDAC2 is commonly expressed in different breast cancer cells we tested. Furthermore, the flow cytometry results showed that the membrane expression of PD-L1 was the highest in MDA-MB-231 cells and the lowest in 4T-1 cells (Fig. 1H). Overall, these findings support that HDAC2 and PD-L1 are highly expressed in TNBC, and HDAC2 is significantly correlated with PD-L1 expression.

### HDAC2 knockout inhibited the proliferation, suppressed colony formation, migration, and cell cycle of TNBC cells

**Fig. 2:**
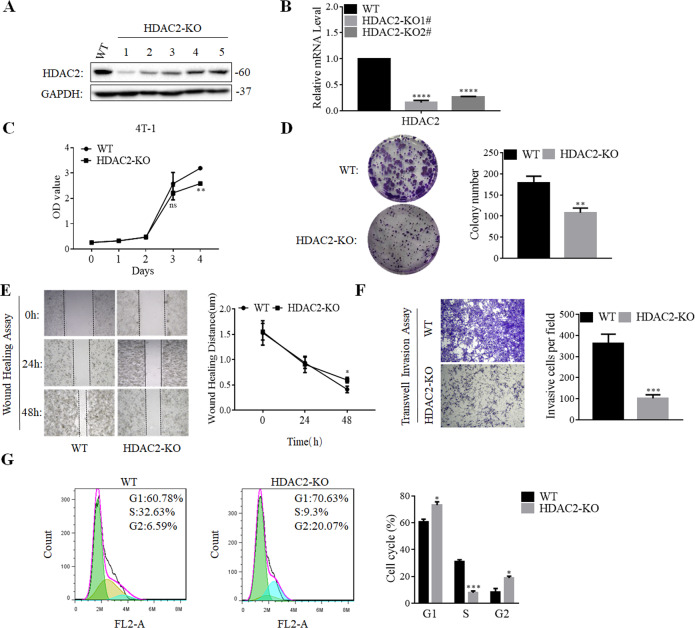
HDAC2 knockout inhibited the proliferation, suppressed colony formation, migration, and cell cycle of TNBC cells. **A**, **B** Western blotting (**A**) and RT-PCR (**B**, *n* = 3) detected the levels of HDAC2 expression in WT cells and HDAC2-KO clones. **C**, **D** Cells (WT, HDAC2-KO) viability and colony formation were tested using the CCK-8 (**C**) and colony-formation analysis (**D**), respectively (*n* = 3). **E**, **F** The wound healing (**E**) and Transwell assay (**F**) results showed the migration of WT and HDAC2-KO cells (*n* = 3). **G** Flow cytometry analysis showed the cell cycle of WT and HDAC2-KO cells (*n* = 3).

To further explore whether HDAC2 affected the biological functions of breast cancer cells, we used CRISPR/Cas9 gene editing method to construct a mouse TNBC cell strains (4T-1) that stably knocked out HDAC2 (HDAC2-KO). The western blotting and RT-PCR analysis showed that the HDAC2 expression in knockout cells was significantly reduced than WT cells, indicating that we have successfully established the HDAC2-KO cells (Fig. 2A, B). Next, we studied the effect of HDAC2 knockout on the malignant behaviors of breast cancer cells by carrying out CCK-8, colony formation assay, wound healing, Transwell migration and cell cycle analysis. As shown in Fig. 2, the proliferation and colony formation of HDAC2-KO cells was significantly declined compared to that of the WT cells (Fig. 2C, D). Furthermore, the results of wound healing and Transwell assays showed that the HDAC2-KO markedly inhibited TNBC cells’ migration (Fig. 2E, F). In addition, we analyzed the cell cycle profiles using flow cytometry method. Figure 2G shows an increase in G1/G2-phase cells and a concomitant decrease in S-phase cells in HDAC2-KO cells when compared with the WT cells. Collectively, the above results reveal that HDAC2 knockout can inhibit cell proliferation, suppress colony formation, migration, and prompt G1/G2 cell cycle arrest of breast cancer cells.

### HDAC2 promoted the IFNγ-induced PD-L1 expression in TNBC cells via activation of the JAK-STAT1 pathway

**Fig. 3:**
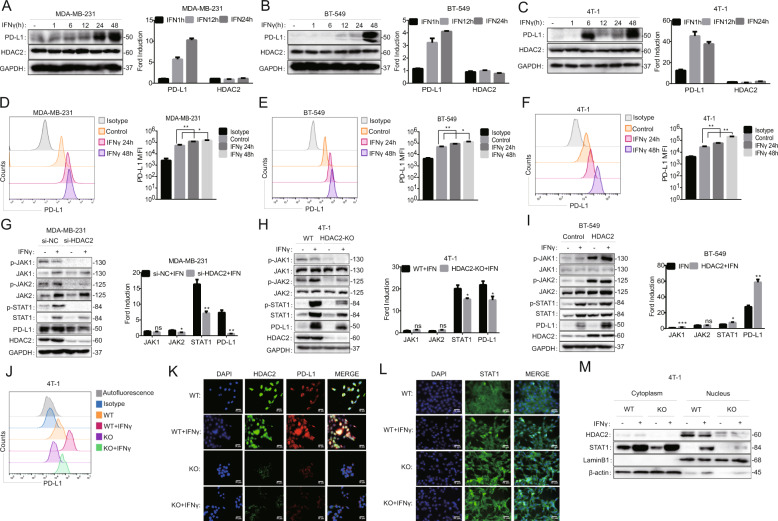
HDAC2 promoted the IFNγ-induced PD-L1 expression in TNBC cells via activation of the JAK–STAT1 pathway. **A**–**C** MDA-MB-231 (**A**), BT-549 (**B**), and 4T-1 (**C**) cells were cultured with IFNγ (100U/ml) for 1, 6, 12, 24, and 48 h. The expression of HDAC2 and PD-L1 was tested by western blotting and RT-PCR (*n* = 3). **D**–**F** MDA-MB-231 (**D**), BT-549 (**E**), and 4T-1 (**F**) cells were cultured with IFNγ (100 U/ml) for 24 and 48 h. The expression of PD-L1 was tested by flow cytometry (*n* = 3). **G** MDA-MB-231 cells were transfected with si-RNA for 24 h, and then cultured with or without IFNγ (100 U/ml) for 24 h. The expression of PD-L1 and HDAC2 was examined by western blotting and RT-PCR (*n* = 3), including the proteins involved in IFNγ pathway. **H** Similar to G, but 4T-1 (WT, HDAC2-KO) cells were stimulated by IFNγ (100 U/ml) for 24 h. **I** Similar to **G**, but BT-549 cells were transfected with HDAC2 expression plasmids for 24 h before IFNγ (100 U/ml) treatment. **J** PD-L1 expression was determined in 4T-1 (WT, HDAC2-KO) by flow cytometry after culturing with or without IFNγ (100 U/ml) for 24 h (*n*=3). **K** The expression of PD-L1 and HDAC2 was examined by immunofluorescence staining in 4T-1 cells (WT, HDAC2-KO) cells after stimulation with or without IFNγ (100 U/ml) for 24 h, original magnification: 100×. Scale bars, 50 μm; *n* = 3 independent experiments were performed with similar results. **L** Similar to **K**, 4T-1 cells (WT and HDAC2-KO) were treated by IFNγ (100 U/ml) for 30 min. The expression of STAT1 was determined by immunofluorescent staining assays. **M** Western blotting results showed the cytoplasmic and nuclear expression of HDAC2 and STAT1 in 4T-1 cells (WT, HDAC2-KO) after culturing with or without IFNγ (100 U/ml) for 12 h.

IFNγ was the strongest inducer of PD-L1 expression in tumor microenvironment. Therefore, to test the effect of IFNγ on PD-L1 induction in TNBC cells, we detected expression of PD-L1 and HDAC2 by using western blotting, RT-PCR, and flow cytometry after the TNBC cells were treated with IFNγ for different times. Western blotting and RT-PCR results showed that IFNγ was able to elevate PD-L1 expression in all TNBC cells we tested, but had no influence on the HDAC2 expression (Fig. 3A–F). To further elucidate the effects of HDAC2 on the PD-L1 induction, we performed HDAC2 gene knockdown or overexpression by transfecting siRNAs specific for HDAC2 or HDAC2 expression plasmids to TNBC cells. The siRNA transfection effectively downregulated the expression of HDAC2 (Fig. S2A). Following the knockdown, the mRNA expression of PD-L1 induced by IFNγ was significantly reduced in HDAC2 siRNA-treated MDA-MB-231 cells compared to the cells transfected with nontargeted control sequences (Fig. S2B). Based on the interference efficiency, we continue the followed experiments by using the No.3 siRNA. Western blotting showed that HDAC2 knockdown also significantly reduced the PD-L1 induction in MDA-MB-231 and BT-549 cells (Fig. S2C). These results indicated that HDAC2 knockdown marginally affected the basal expression of PD-L1, but can decrease the PD-L1 upregulation upon stimulation with IFNγ. Of note, the PD-L1 induction was further enhanced by HDAC2-overexpression in TNBC cell lines (Fig. S2D–F). The above conclusion was strengthened in studies of 4T-1 cells in which HDAC2 was stably depleted (Fig. S2G).

Since IFNγ-induced gene expression relies on robust signaling transduction, we asked whether HDAC2 is involved in the IFNγ–JAK/STAT1 pathway. Consequently, we tested the changes of phosphorylation of JAK1 (*p*-JAK1), JAK2 (*p*-JAK2), and STAT1 (*p*-STAT1) after HDAC2 knockdown or overexpression by using western blotting analysis. As shown in Fig. 3, IFNγ treatment promptly caused JAK1, JAK2, and STAT1 phosphorylation in MDA-MB-231 and 4T-1 cells, but not in BT-549 cells, followed by the upregulation of STAT1 and PD-L1 expression in all three TNBC cells we tested (Fig. 3G–I). In contrast, the raised *p*-JAK1, *p*-JAK2, and *p*-STAT1 induced by IFNγ was inhibited by HDAC2 knockdown in MDA-MB-231 cells (Fig. 3G). We further confirmed this effect in the HDAC2-KO cells (Fig. 3H). Furthermore, the overexpression of HDAC2 promoted the levels of *p*-JAK1, *p*-JAK2 and *p*-STAT1 in the presence or absence of IFNγ in BT-549 cells (Fig. 3I). In addition, flow cytometry analysis showed that the increased PD-L1 expression of cell surface induced by IFNγ was also declined in HDAC2-KO cells compared to the WT cells (Fig. 3J, Fig. S2H). Because the STAT1 homodimerized and translocated from the cytoplasm to the nucleus followed by phosphorylation, which were important steps of the IFNγ signal transduction. Thus, we next used immunofluorescence staining to analyze the effect of HDAC2 on PD-L1 induction and STAT1 nuclear transduction. As shown in Fig. 3K and L, HDAC2 knockout inhibited the intracellular PD-L1 expression, as well as the translocation of STAT1 to the nucleus stimulated by IFNγ. This effect was further confirmed in the detection of nuclear and plasma protein expression by western blotting (Fig. 3M). Overall, all these data suggest that HDAC2 promotes the IFNγ-induced PD-L1 expression in TNBC cells via activation of JAK–STAT1 pathway.

### HDAC2 knockout decreased IFNγ-induced STAT1 recruitment and histone acetylation of PD-L1 promoter

**Fig. 4:**
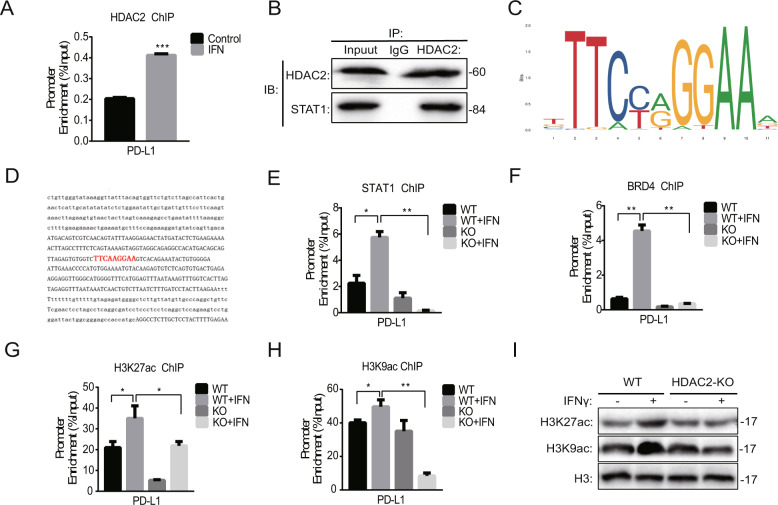
HDAC2 knockout decreased IFNγ-induced STAT1 recruitment and histone acetylation of PD-L1 promoter. **A** ChIP-qPCR was performed after 4T-1 cells (WT, HDAC2-KO) were treated with or without IFNγ (100 U/ml) for six hours (*n* = 3). **B** 4T-1 (WT, HDAC2-KO) cells were cultured with IFNγ (100 U/ml) for 24 h, and western blotting was performed after cells were precipitated with antibodies of HDAC2 or IgG. **C**, **D** The sequence (**C**) and position (**D**) of the binding sites of STAT1 in PD-L1 promoter. **E**–**H** ChIP-qPCR was performed using STAT1 (**E**), BRD4 (**F**), H3K27ac (**G**), and H3K9ac (**H**) antibodies after 4T-1 cells (WT, HDAC2-KO) were treated with or without IFNγ (100 U/ml) for six hours (*n* = 3). **I** Western blotting was conducted after 4T-1 (WT, HDAC2-KO) cells were stimulated with or without IFNγ (100 U/ml) for 12 h.

HDACs regulate gene expression mainly through the recruitment to the gene promoter [34]. Therefore, we further tested the recruitment of HDAC2 to PD-L1 promoter by performing ChIP-qPCR assay. As expected, the binding level of HDAC2 with PD-L1 promoter was increased compared to the basal level after treatment with IFNγ, indicating that HDAC2 was recruited to the PD-L1 promoter (Fig. 4A). Previous reports documented that the STAT1 was recruited and bound with HDAC2 to regulate IFN-induced gene expression [35]. Using a co-immunoprecipitation assay, we confirmed the interaction of IFNγ-activated STAT1 with HDAC2 (Fig. 4B).

According to previous reports, the key step of IFNγ-induced gene transcription depends on the binding of STAT1 with gamma interferon activation site (GAS) and subsequently recruits HAT and HDAC to induce histone hyperacetylation and chromatin remodeling [36]. Moreover, bromodomain-containing 4 (BRD4) was rapidly recruited to the PD-L1 locus, accompanied by increased H3K27ac and RNA Polymerase II (RNA Pol II) occupancy in melanoma cells after IFNγ stimulation [37]. It has been reported that H3K9 acetylation also plays a positive role in the expression of PD-L1 [38]. We, therefore, tested whether HDAC2 knockout was involved in IFNγ-induced histone acetylation and transcription factor recruitment on PD-L1 promoter through ChIP-qPCR assay. First, we analyzed the sequence of PD-L1 promoter using JASPAR and ECR browser, and found the putative binding sites for STAT1 at the position between −1163 and −1173 bp upstream from TSS (Fig. 4C, D). We further conducted ChIP-qPCR with primers covering the binding sites of STAT1 in PD-L1 promoter in 4T-1 cells. Figure 4E shows that the IFNγ treatment increased STAT1 occupancy at the PD-L1 promoter, and this binding was decreased after HDAC2 knockout. In addition, HDAC2-KO also reduced IFNγ-induced recruitment of BRD4 to the PD-L1 promoter (Fig. 4F). Consistent with the previous study, the enrichment of H3K27 and H3K9 acetylation (H3K27ac, H3K9ac) in PD-L1 promoter, the marker of active transcription, was upregulated by IFNγ stimulation. However, HDAC2-KO reduced the upregulation of H3K27ac and H3K9ac induced by IFNγ at the same site of PD-L1 promoter, as well as these two proteins expression (Fig. 4G–I). These results suggest that HDAC2 activates PD-L1 expression by facilitating IFNγ-induced STAT1 and BRD4 recruitment. Furthermore, the IFNγ-induced H3K27 and H3K9 acetylation of PD-L1 promoter was also inhibited by HDAC2 knockout.

### HDAC2 knockout impaired tumor growth and PD‑L1 production in vivo

**Fig. 5:**
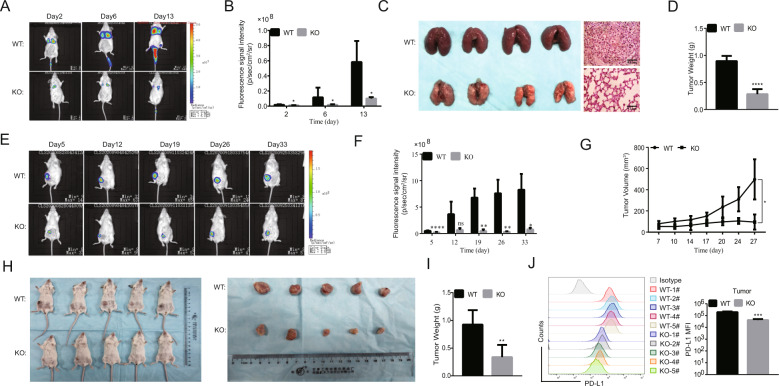
HDAC2 knockout impaired tumor growth and PD–L1 production in vivo. **A** Representative whole-body luminescent images of mice taken at different times after WT and HDAC2-KO cells were intravenously injected into Balb/c mice. **B** Average lung signal intensities of five mice-injected cells. The measurements were performed at the indicated time points (*n* = 5). **C, D** The mice were sacrificed at the 13th day after cell injection, and tumor growth was monitored by gross morphology, H&E staining (**C**), and lung weight (**D**, *n* = 5). **E**, **F** Similar to **A** and **B**, but 3 × 10^6^ cells were implanted into the breast fat pad of Balb/c mice. **G** Tumor volumes were calculated every 3–4 days after tumor cells’ implantation (*n* = 5). **H**, **I** Similar to **C** and **D**, but the mice were sacrificed at 33 days after tumor cells’ inoculation (*n* = 5). **J** PD-L1 expression was determined in tumors by flow cytometry (*n* = 5).

Previous study has shown that the knockdown of PD-L1 reduces tumor growth and metastasis [7, 9]. We therefore employed two tumor models for in vivo research, including lung metastasis model and orthotropic tumor model. To explore the effect of upregulation of PD-L1 by HDAC2 in the metastasis of TNBC cells, we respectively injected control cells (WT) and HDAC2-knockout cells (HDAC2-KO) into Balb/c and Balb/c-nu mice via the tail vein and monitored the growth of lung tumors by IVIS system. As shown in Fig. 5A, B, the lung metastasis models were successfully established in those mice injected WT cells and the size of the lung tumors was larger than that of mice injected HDAC2-KO cells. We sacrificed the mice on the 13th day after tumor cells injection. Surprisingly, there was a strong reduction in the tumor formation or no tumor growth in the mice injected HDAC2-KO cells. These results were further confirmed by H&E staining and weight of lung tissues (Fig. 5C, D). However, the effect of HDAC2-KO was largely reversed in immunocompromised nude mice (Fig. S3A, B), supporting the notion that the antitumor effect mainly depends on T cells. Next, to assess the effect of HDAC2 on the tumor growth, the mammary fat pad injection was performed in BALB/c mice with WT and HDAC2-KO cells, and the growth was also monitored by IVIS system. Mice were sacrificed on day 33. We found that mice implanted with HDAC2-KO cells developed smaller tumors than those implanted with WT cells (Fig. 5E–I). Similar results were obtained on another HDAC2-KO clone (clone 2#) cell in vivo experiment (Fig. S3C). Next, we analyzed the cell surface expression of PD-L1 in tumor tissues by flow cytometry. As shown in Fig. 5J, compared with the WT group, the HDAC2-KO group had a lower expression of PD-L1. On the other hand, the detection results for protein and mRNA confirmed that the expression of PD-L1 in HDAC2-KO tumor tissues was significantly reduced than WT group (Fig. S3D, E). We also examined Ki-67 and PD-L1 expression in the orthotopic tumors of two groups through immunohistochemistry (IHC) staining. The representative images of tumors were shown in Fig. S3F, HDAC2-KO group exhibited a significantly decreased expression of PD-L1 and Ki-67 compared with the WT group. Thus, these findings reveal that the HDAC2 contributes to the tumor growth and lung metastasis. Besides, as we hypothesized, the HDAC2-KO impaired PD-L1 production, and hence the PD-L1 reduction was maintained in vivo.

### HDAC2 knockout promoted tumor lymphocyte infiltration

**Fig. 6:**
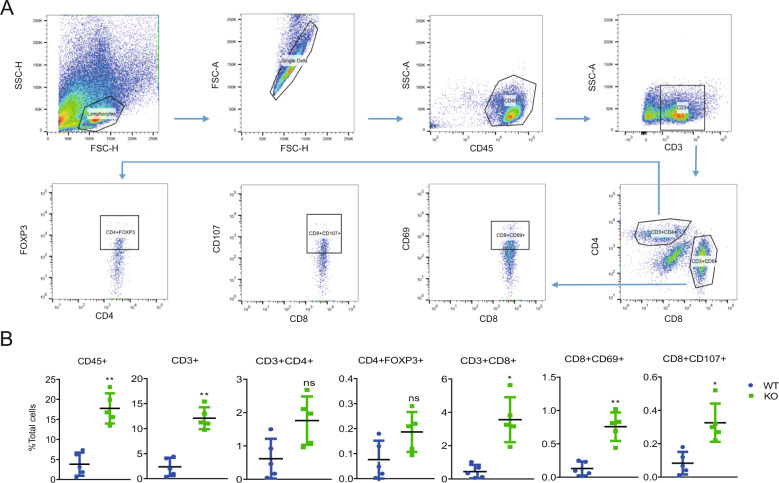
HDAC2 knockout promoted tumor lymphocyte infiltration. **A**, **B** The Balb/c mice were sacrificed at 33 days after WT and HDAC2-KO cells’ (4T-1) inoculation, and tumors of each group were isolated, and the infiltration of lymphocytes was analyzed by flow cytometry using the indicated antibodies. Gating strategy (**A**) and representative plots (KO group) are shown. Proportions of CD45+, CD3+, CD3+CD4+, CD3+CD4+FOXP3+, CD3+CD8+, CD3+CD8+CD69+, and CD3+ CD8+CD107+ T cells in tumors of each group (**B**, *n* = 5).

HDAC2 and PD-L1 have been reported to have both pro-tumorigenesis and immunomodulatory effects, which contributes to the formation of tumor-immunosuppressive microenvironment [30]. We therefore investigated the effect of HDAC2-KO on the T-lymphocyte infiltration. We isolated tumor tissues from the above mouse models and detected some molecular markers of T lymphocytes by flow cytometry technique. As shown in Fig. 6, compared to tumor from WT group, the HDAC2-KO group had a higher percentage of CD45+ lymphocytes, CD3+, and CD8+ T cells, but not CD4+ population or CD4+FOXP3+ Treg cells. Moreover, the percent of cytotoxic T cells (CD8+CD69+, CD8+CD107+ T cells) was significantly elevated in HDAC2-KO group (Fig. 6A, B). Overall, these results indicate that the HDAC2-KO changed the T-lymphocyte proportions of tumors and reduced the immunosuppression of tumor-bearing mice.

## Discussion

TNBC remains an extremely challenging disease due to the immune escape, metastasis, and lack of effective targeted therapies. To date, numerous studies have been carried out to uncover the potential therapeutic targets of TNBC. Among them, the overexpression of PD-L1 is a promising biomarker for the treatment of TNBC [39]. The specific antibodies for PD-1/PD-L1 pathway lead to stronger tumor regression in vivo, and some clinical trials have demonstrated promising results in TNBC [11]. It was reported that the PD-L1 expression is higher in TNBC than other subtypes of breast cancer, and our results were consistent with this conclusion. In the current study, we identified that PD-L1 and HDAC2 were overexpressed in TNBC, and there was a significant correlation between those two genes from the breast cancer online dataset. Our data provided a novel mechanism of HDAC2 in PD-L1 regulation via the activation of the IFNγ–JAK/STAT signaling pathway and chromatin remodeling. In addition, HDAC2 knockout retarded tumor growth, metastasis, and decreased PD-L1 production in vivo. These results have remarkably enriched our understanding toward the role of HDAC2 in breast cancer development and PD-L1 expression, suggesting a way for enhanced antitumor immunity when targeting the HDAC2 in TNBC.

The molecular mechanism driving PD-L1 overexpression of TNBC remains to be determined. In breast cancer, it was reported that the expression of PD-L1 was mainly regulated by IFNγ via the JAK/STAT pathway [17]. Other underlying mechanisms may also contribute to PD-L1 regulation, including the loss of PTEN and the ensuing activation of the PI3K pathway [13]. Here, we focused on the mechanism of PD-L1 induction by IFNγ. HDAC inhibitors (HDACi) have been reported to prevent IFNγ–JAK/STAT signaling pathway and STAT1-dependent gene activation [28]. Therefore, we asked whether HDAC2 was also involved in the PD-L1 induction by IFNγ. As an important epigenetic modifier, HDAC2 has been identified as an activator of IFN-induced STAT1-dependent gene expression [40]. Here, we demonstrated that HDAC2 promotes IFNγ signaling by upregulating the phosphorylation of JAK1, JAK2, and STAT1, as well as the translocation of STAT1 to the nucleus and the recruitment of STAT1 to the promoter of PD-L1. Our results also demonstrated that HDAC2 was recruited to the PD-L1 promoter by STAT1 after IFNγ treatment. Besides, previous studies reported that a phosphoacetyl switch regulates STAT1 signaling and HDACi treatment led to the hyperacetylation of STAT1, which restrained its phosphorylation and activation [41, 42]. However, this conclusion is still controversial due to the results that were unable to be repeated by other researchers [43]. Other investigators documented that the impairment of IFNγ signaling mediated by HDACi was related to the downregulation of IFNGR [44] expression or upregulation of negative-feedback gene expression of IFNγ pathway, such as SOCS [45]. Herein, we did not find the effect of HDAC2 on the expression of those factors (data not shown), but our data showed that the JAK and STAT1 expression were increased by HDAC2 overexpression, which indicated the HDAC2’s role in transcriptional regulation of JAK/STAT1. In addition, H3K27, H3K9 acetylation and BRD4 recruitment were shown to alter the chromatin status and promoted transcription of PD-L1 in cancer cells [37, 38]. In this study, IFNγ treatment induced the H3K27, and H3K9 acetylation and was accompanied by the recruitment of BRD4 at the PD-L1 promoter, but HDAC2 knockout inhibited this process, suggesting that HDAC2 is required for the chromatin remodeling of IFNγ-induced PD-L1 expression. Previous studies have investigated the regulation by specific HDACs on the PD-L1 expression of tumor cells, including HDAC6 and HDAC8, but the results are not very consistent. In particular, HDAC6 appears to have both positive and negative effects in different cancers [25, 46, 47]. These contradicting conclusions may derive from distinct experiment models and systems involved. Some researchers reported the PD-L1 expression was increased by treatment of pan-HDAC inhibitors in breast cancer cells. In that study, neither single nor a combination of HDAC1, HDAC2, and HDAC3 silencing resulted in increased transcription of PD-L1 in MDA-MB-231 cells [23]. This indicates that one single HDAC does not affect the basal level of PD-L1 expression. Our results also supported this conclusion and suggested that certain HDAC2 took part in regulating the IFNγ-induced PD-L1 expression. Importantly, recent studies have reported that the deacetylation of PD-L1 by HDAC2 triggers its nuclear accumulation, which facilitates the evasion of tumor cells from the immune surveillance during the metastatic process. Blocking the nuclear translocation of PD-L1 with HDAC2-specific inhibitor decreased some immune checkpoint genes’ transcription, resulting in increased CD8+ T-cell infiltration in tumors, which in turn augments the antitumor effect of anti-PD-1 treatment in vivo [30]. Consistently, besides the regulation of PD-L1 function, our results further confirmed that HDAC2 also played a positive role in the PD-L1 induction. Nevertheless, our study did not explore in more depth and the underlying mechanisms need to be confirmed by further experiments.

As the most important deacetylases involved in epigenetic regulation, HDAC2 is closely associated with the occurrence and development of cancers [48, 49]. More recently, the overexpression of HDAC2 was found to be correlated with tumor EMT process [50], metastasis [51], higher Ki-67 level, and multidrug-resistance protein expression in breast cancer [52]. HDAC2 knockdown can inhibit MDA-MB-231 cells’ migration [53], and promote MCF-7 cells’ aggressiveness via enhancing the Nav1.5 expression [54]. Here, our data demonstrate that HDAC2 promotes the proliferation, migration, and cell cycle of 4T-1 cells, suggesting that HDAC2 acts as an oncogene in TNBC. Consistently, high HDAC2 expression is correlated with the poor prognosis of breast cancer patients. In addition, HDAC2 is essential for T-cell development and affects cytokine signaling involved in immune response [55, 56]. For instance, HDAC2 degrades CIITA to antagonize its activity and promotes the expression of MHC-II [57], highlighting its important role in the anti-tumor reactivity. Our results indicate that HDAC2 also has a role in immune evasion of breast cancer cells. In support of the result, HDAC2 knockout reduced the PD-L1 expression, promoted tumor lymphocyte infiltration, inhibited tumor growth, and metastasis in mouse models. Whether this effect is related to the inhibition of breast cancer progression by T cells remains to be tested. From our point of view, HDAC2 may exacerbate immunosuppression by upregulating tumor PD-L1 expression, suggesting that HDAC2 is a promising therapeutic target to control tumor progression, and the development of anticancer drugs specific for HDAC2 may inhibit tumor growth and immune escape of cancers. Further researches of HDAC2-specific inhibitors or combined PD-1/PD-L1 antibodies in the treatment of TNBC are clearly warranted.

## Supplementary information

Supplementary Materials

Supplementary tables

Supplementary Figures

Figure S1

Figure S2

Figure S3

## Data Availability

The datasets used and/or analyzed during the current study are available from the corresponding author on reasonable request.

## Abbreviations

TNBC: triple-negative breast cancer
ER: estrogen receptor
PR: progesterone receptor
HER2/neu: human epidermal growth factor receptor 2
PD-1: programmed cell death-1
PD-L1: programmed cell death-Ligand 1
TCR: T cell receptor
PTPN11: protein tyrosine phosphatase nonreceptor type 11
APCs: antigen-presenting cells
PTEN: phosphatase and tensin homolog deleted on chromosome ten
PI3K: phosphoinositide 3-kinase
IFNγ: interferon gamma
JAK: janus kinase
STAT: signal transducer and activator of transcription
HDAC2: histone deacetylase 2
HAT: histone acetyltransferase
HOXA5: homeobox A5
GAS: gamma-interferon activation site
H3K27ac: acetylated H3 lysine 27
H3K9ac: acetylated H3 lysine 9
BRD4: bromodomain containing 4
SOCS: suppressor of cytokine signaling
CIITA: class II major histocompatibility complex transactivator
MHC-II: major histocompatibility complex, class II
ChIP: chromatin immunoprecipitation
