# Supplementary material for: Histone deacetylase 2 knockout suppresses immune escape of triple-negative breast cancer cells via downregulating PD-L1 expression
Source: Cell Death Dis. 2021 Aug 7;12(8):779. doi: 10.1038/s41419-021-04047-2 (PMC8349356; doi:10.1038/s41419-021-04047-2)
Supplement: Supplementary file 1 — Supplementary Materials [file 41419_2021_4047_MOESM1_ESM.docx]

**Supplementary Materials:**

**Antibodies and Reagents list:**

| Antibodies/Reagents | Source | Identifier | Usage | Working dilution |
| --- | --- | --- | --- | --- |
| Recombinant Human IFNγ | Biolegend | #570206 | Treatment | 100U/ml |
| Recombinant Mouse IFNγ | Biolegend | #575306 | Treatment | 100U/ml |
| Anti-human PD-L1 (29E.2A3) PE | Biolegend | #329705 | FCM | Ready-to-use |
| Anti-mouse PD-L1 (10F.9G2) PE | Biolegend | #124305 | FCM | Ready-to-use |
| Anti-Rat IgG2b, κ Isotype Ctrl (RTK4530) PE | Biolegend | #400607 | FCM | Ready-to-use |
| Anti-IgG2b, κ Isotype Ctrl (MPC-11) PE | Biolegend | #400311 | FCM | Ready-to-use |
| Anti-human PD-L1 (E1L3N) | CST | #13684 | WB | 1:1000 |
| Anti-mouse PD-L1 (EPR20529) | Abcam | #ab213480 | WB/IF | 1:1000/ 1:50 |
| Anti-PD-L1 | Abcam | #ab233482 | WB/IHC | 1:1000/ 1:200 |
| Anti-HDAC2 (D6S5P) | CST | #57156 | WB/IF/ChIP | 1:1000/ 1:400/ 1:50 |
| Anti-HDAC2 (Y461) | Abcam | #ab32117 | WB/IHC | 1:2000/ 1:200 |
| Anti-Flag (FG4R) | Abcam | #ab125243 | WB | 1:1000 |
| Anti-GAPDH (1E6D9) | Proteintech | #60004-1-Ig | WB | 1:10000 |
| Anti-rabbit IgG, HRP-linked Antibody | CST | #7074P2 | WB | 1:2000 |
| Propidium Iodide | Thermo Fisher | #P1304MP | FCM | Ready-to-use |
| RNase A | Thermo Fisher | #R1253 | FCM | Ready-to-use |
| Anti-Mouse IgG (H+L) Highly Cross-Adsorbed Secondary Antibody, Alexa Fluor Plus 488 | Invitrogen | #A32723 | IF | Ready-to-use |
| Anti-Rabbit IgG (H+L) Cross-Adsorbed Secondary Antibody, Alexa Fluor 488 | Invitrogen | #A-11008 | IF | Ready-to-use |
| Anti-Mouse IgG (H+L) Highly Cross-Adsorbed Secondary Antibody, Alexa Fluor Plus 594 | Invitrogen | #A32754 | IF | Ready-to-use |
| Anti-Rabbit IgG (H+L) Cross-Adsorbed Secondary Antibody, Alexa Fluor 594 | Invitrogen | #A11012 | IF | Ready-to-use |
| Anti-JAK1 (E3A6M) | CST | #29261 | WB | 1:1000 |
| Anti-p-JAK1 (Tyr1034/1035) | CST | #3331 | WB | 1:1000 |
| Anti-JAK2 (E4Y4D) | CST | #74987 | WB | 1:1000 |
| Anti-p-JAK2 (Tyr1007/1008) | CST | #3771 | WB | 1:1000 |
| Anti-STAT1 (D1K9Y) | CST | #14994 | WB/IF/ChIP | 1:1000/ 1:400/ 1:50 |
| Anti-p-STAT1 (Tyr701) | CST | #9167 | WB | 1:1000 |
| DAPI | Invitrogen | # S36973 | IF | Ready-to-use |
| Anti-LaminB1 (D9V6H) | CST | #13435 | WB | 1:1000 |
| Anti-β-actin (8H10D10) | CST | #3700 | WB | 1:1000 |
| Anti-H3K27ac (D5E4) | CST | #8173 | ChIP/WB | 1:50/ 1:1000 |
| Anti-H3K9 ac (C5B11) | CST | #9649 | ChIP/WB | 1:50/ 1:1000 |
| Anti-H3 (D1H2) | CST | #4499 | WB | 1:2000 |
| Anti-BRD4 (E2A7X) | CST | #13440 | ChIP | 1:50 |
| Anti-Normal Rabbit IgG | CST | #2729 | ChIP | 1:50 |
| Anti-Ki67 | Proteintech | #27309-1-AP | IHC | 1:2000 |
| HRP-congested second antibody | CST | #8125 | IHC | Ready-to-use |
| DAB | CST | #8059 | IHC | Ready-to-use |
| D-Luciferin Potassium Salt | PerkinElmer | #122799 | Living image | 10 mg/kg |
| Anti-mouse CD16/32 (93) | Biolegend | #101319 | FCM | Ready-to-use |
| Anti-mouse CD45 (QA17A26)  APC/Cyanine7 | Biolegend | #157617 | FCM | Ready-to-use |
| Anti-mouse CD3 (17A2) PE/Cyanine7 | Biolegend | #100219 | FCM | Ready-to-use |
| Anti-mouse CD4 (GK1.5) Alex Flour 647 | Biolegend | #100426 | FCM | Ready-to-use |
| Anti-mouse FOXP3 (MF-14) Brilliant Violet 421 | Biolegend | #126419 | FCM | Ready-to-use |
| Anti-mouse CD8 (53-6.7)  FITC | Biolegend | #100705 | FCM | Ready-to-use |
| Anti-mouse CD69 (H1.2F3) Brilliant Violet 650 | Biolegend | #104541 | FCM | Ready-to-use |
| Anti-mouse CD107a (1D4B)  PE | Biolegend | #121611 | FCM | Ready-to-use |
