# Supplementary material for: Histone deacetylase 2 knockout suppresses immune escape of triple-negative breast cancer cells via downregulating PD-L1 expression
Source: Cell Death Dis. 2021 Aug 7;12(8):779. doi: 10.1038/s41419-021-04047-2 (PMC8349356; doi:10.1038/s41419-021-04047-2)
Supplement: Supplementary file 2 — Supplementary tables [file 41419_2021_4047_MOESM2_ESM.docx]

**Supplementary tables.** Sequences of siRNA and primer used in this study.

**Supplementary table 1.** Primer sequences for RT-PCR

| Gene symbol | Sense primer(5’-3’) | Antisense primer(5’-3’) |
| --- | --- | --- |
| GAPDH(H) | CTCCAAAATCAAGTGGGGCG | TGGTTCACACCCATGACGAA |
| JAK1(H) | ACCACGCTCTGGGAAATCTG | CTGTCATCTGGCCATGGGTT |
| JAK2(H) | TTCAGCCAATGCAAAGCCAC | TCTCGCTCGACAGCAAAAGT |
| STAT1(H) | TCAGGCTCAGTCGGGGAATA | CAGTGAACTGGACCCCTGTC |
| PD-L1(H) | CTGTCACGGTTCCCAAGGAC | GGAGGATGTGCCAGAGGTAGT |
| HDAC2(H) | GCCACTGCCGAAGAAATGAC | TCCAGCCCAATTAACAGCCA |
| GAPDH(M) | CTACCCCCAATGTGTCCGTC | TGAAGTCGCAGGAGACAACC |
| JAK1(M) | TCCTGAGGGAGACAACACAG | ACATGGCAGACGCTTTCCTT |
| JAK2(M) | GTGGAGATGTGCCGCTATGA | GCCTTGGCAATCTTCCGTTG |
| STAT1(M) | TGGGCGTCTATCCTGTGGTA | TGAATGTGATGGCCCCTTCC |
| PD-L1(M) | GGGCGTTTACTATCACGGCT | AGGGCAGCATTTCCCTTCAA |
| HDAC2(M) | TTTTGTCAGCTCTCCACGGG | GAAACAACCAAGCCTGTCCC |

**Supplementary table 2.** The sequences of siRNA

| siRNA | Sense primer(5’-3’) | Antisense primer(5’-3’) |
| --- | --- | --- |
| si-NC | UUCUCCGAACGUGUCACGUTT | ACGUGACACGUUCGGAGAATT |
| si-HDAC2-1# | CCCAUAACUUGCUGUUAAATT | UUUAACAGCAAGUUAUGGGTT |
| si-HDAC2-2# | GCAAAUACUAUGCUGUCAATT | AUAUUCUGGAGUGUUCUGGTT |
| si-HDAC2-3# | CCAGAACACUCCAGAAUAUTT | GCCTCGTTTTGCCCTTTGAG |

**Supplementary table 3.** Primer sequences for ChIP-qPCR

| Gene symbol | Sense primer(5’-3’) | Antisense primer(5’-3’) |
| --- | --- | --- |
| PD-L1(STAT1 binding site) | AGGGATGGATTTGAGCACAGG | GACGCTCATTCAAGATGGGGA |
| PD-L1 | GTTCACAAAGGGCACGGTTC | GCCCTTTCTGGATACGTGGA |
