# Supplementary material for: Histone deacetylase 2 knockout suppresses immune escape of triple-negative breast cancer cells via downregulating PD-L1 expression
Source: Cell Death Dis. 2021 Aug 7;12(8):779. doi: 10.1038/s41419-021-04047-2 (PMC8349356; doi:10.1038/s41419-021-04047-2)
Supplement: Supplementary file 3 — Supplementary Figures [file 41419_2021_4047_MOESM3_ESM.docx]

**Figure S1**


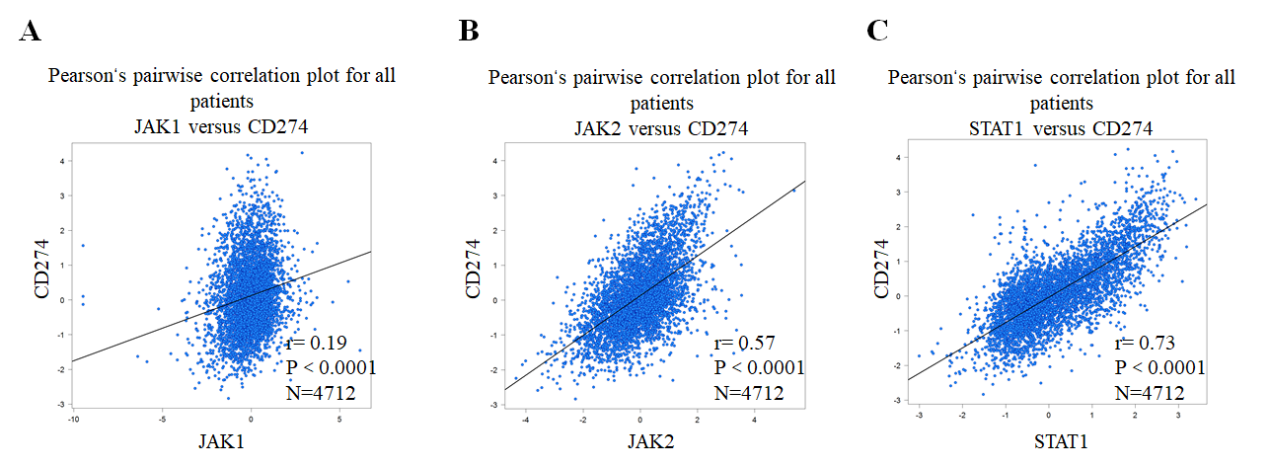


Figure S1. The JAK-STAT1 signaling pathway was positively correlated with PD-L1 expression. **(A-C)** The correlation between JAK1 (A), JAK2 (B), STAT1 (C) and PD-L1 expression was analyzed by bc-GenExMiner v4.5.

**Figure S2**


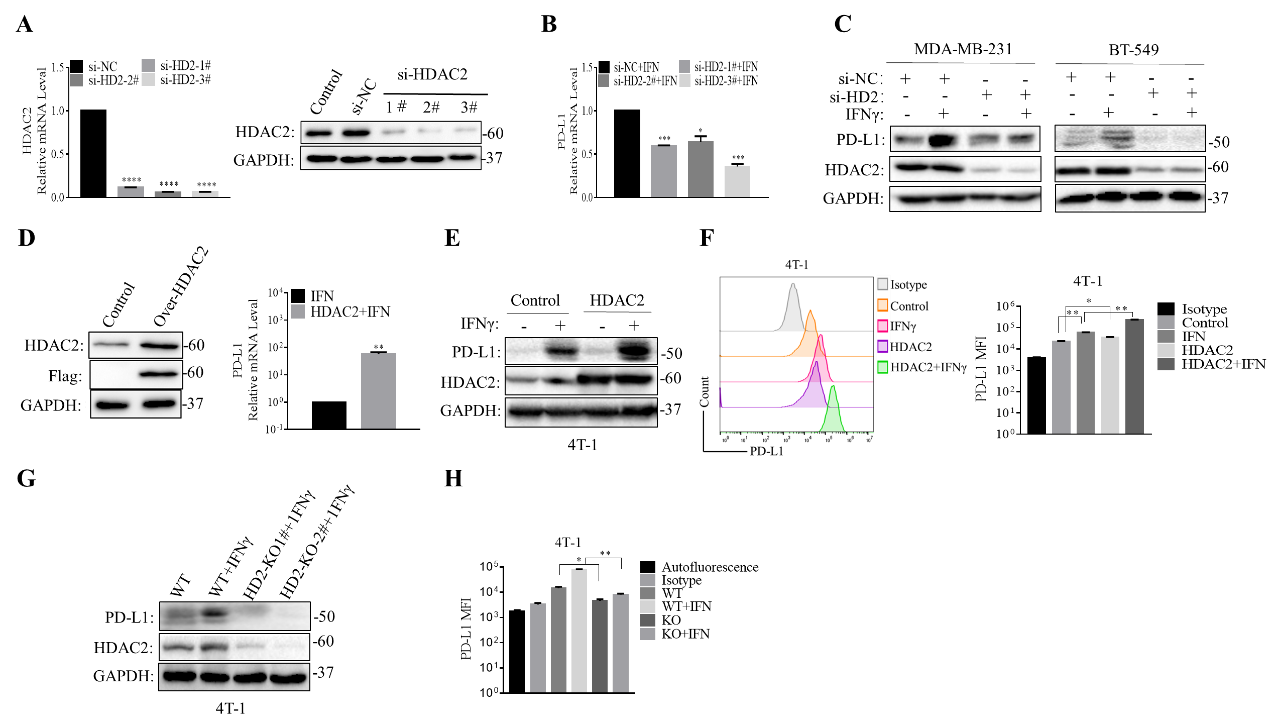


Figure S2. HDAC2 promoted the IFNγ-induced PD-L1 expression in TNBC cells. **(A)** MDA-MB-231 cells were transfected with si-RNA for 48 hours. The expression of HDAC2 was examined by RT-PCR (n=3) and western blotting. **(B)** MDA-MB-231 cells were transfected with si-RNA for 24 hours, and then cultured with or without IFNγ (100U/ml) for 24 hours. The mRNA levels of PD-L1 was tested by RT-PCR (n=3). **(C)** The same method as B was used to detect the protein expression of PD-L1 in MDA-MB-231 and BT-549 cells. **(D)** Similar to B, HDAC2 expression plasmids were used to transfect BT-549 cells for 24 h before IFNγ stimulation, and the transfection efficiency and PD-L1 expression were determined by western blotting and RT-PCR (n=3), respectively. **(E-F)** Similar to D, but the HDAC2 expression plasmids were used to transfect 4T-1 cells, and the PD-L1 expression were determined by western blotting (E) and flow cytometry (F, n=3), respectively. **(G)** 4T-1 cells (WT, HDAC2-KO1#, HDAC2-KO2#) were treated with IFNγ (100 U/ml) for 24 h, and then the PD-L1 expression was examined by western blotting. **(H)** Densitometric analysis of the data in Fig. 3J (n=3).

**Figure S3**


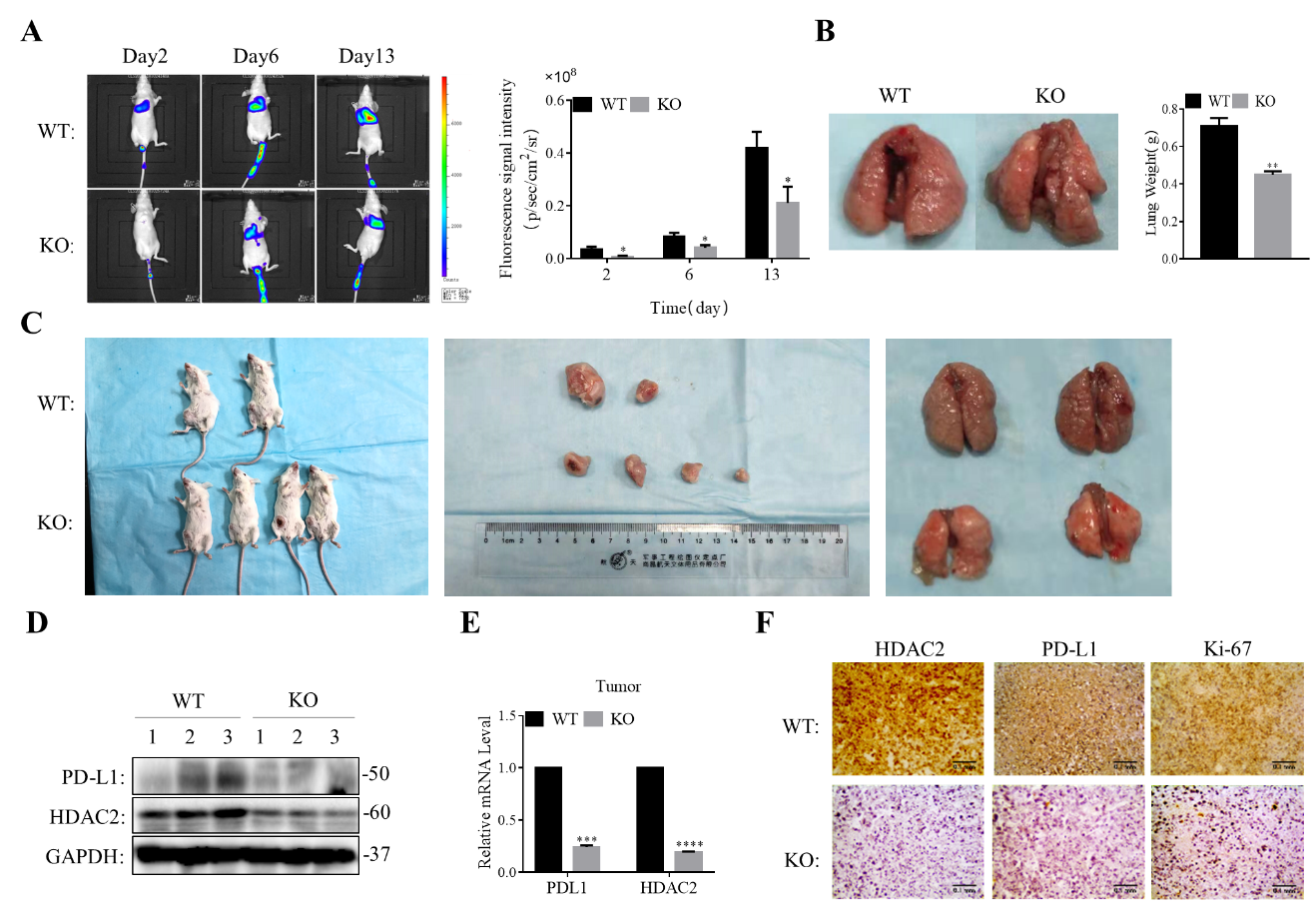


Figure S3. HDAC2 knockout impaired tumor growth and PD‑L1 production in vivo. **(A)** Representative whole body luminescent images of mice taken at different times after WT and HDAC2-KO cells were intravenously injected into Balb/c-nu mice. Average lung signal intensities of five mice injected cells (n=5). The measurements were performed at the indicated time points. **(B)** The mice were sacrificed at 13 days after cells injection, and tumor growth was monitored by gross morphology and lung weight (n=5). **(C)** WT and HDAC2-KO cells were injected into the breast fat pad or vein of Balb/c mice. Representative image of whole body, tumor, and lung tissues were taken at 33th or 13th day after tumor cell injection. **(D, E)** The PD-L1 and HDAC2 expression of tumor tissues were analyzed by western blotting (D) and RT-PCR (E, n=5). **(F)** Immunohistochemistry analysis of HDAC2, PD-L1 and Ki-67 in tumor sections. Scale bars, 0.1 mm.
