# Supplementary figures and images for: Histone deacetylase 2 knockout suppresses immune escape of triple-negative breast cancer cells via downregulating PD-L1 expression
Source: Cell Death Dis. 2021 Aug 7;12(8):779. doi: 10.1038/s41419-021-04047-2 (PMC8349356; doi:10.1038/s41419-021-04047-2)

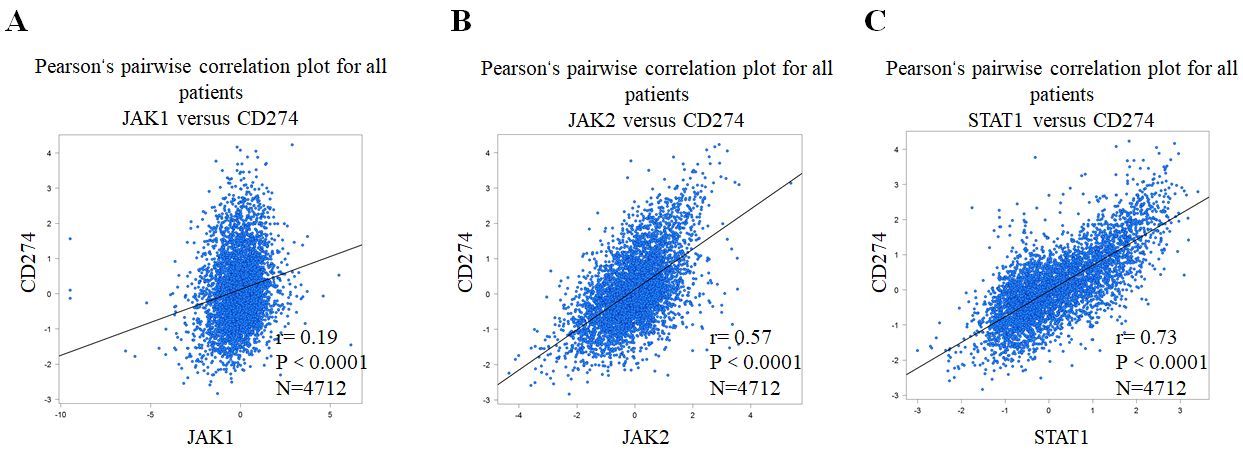

Supplement: Supplementary file 4 — Figure S1 [file 41419_2021_4047_MOESM4_ESM.tif]

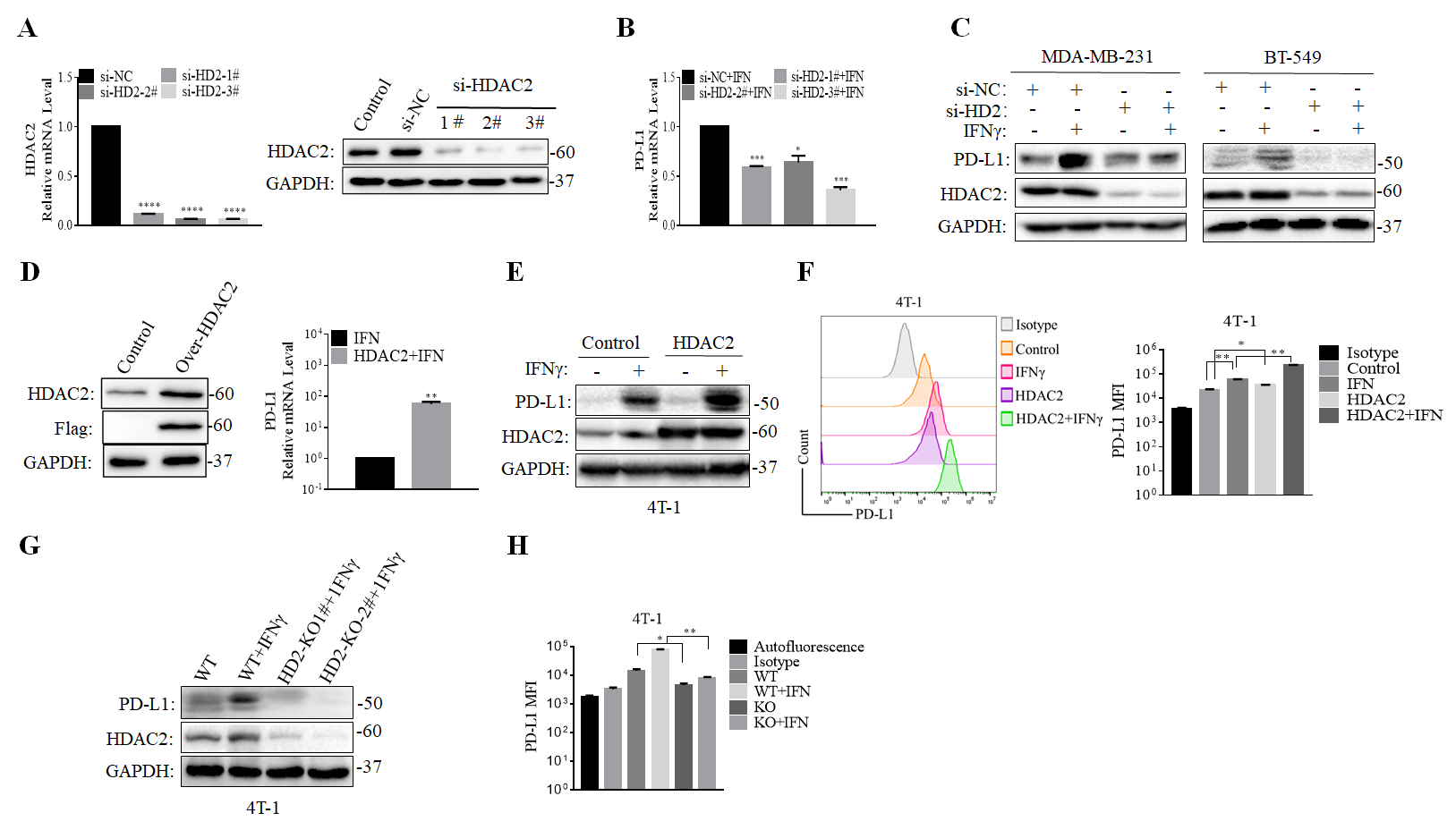

Supplement: Supplementary file 5 — Figure S2 [file 41419_2021_4047_MOESM5_ESM.tif]

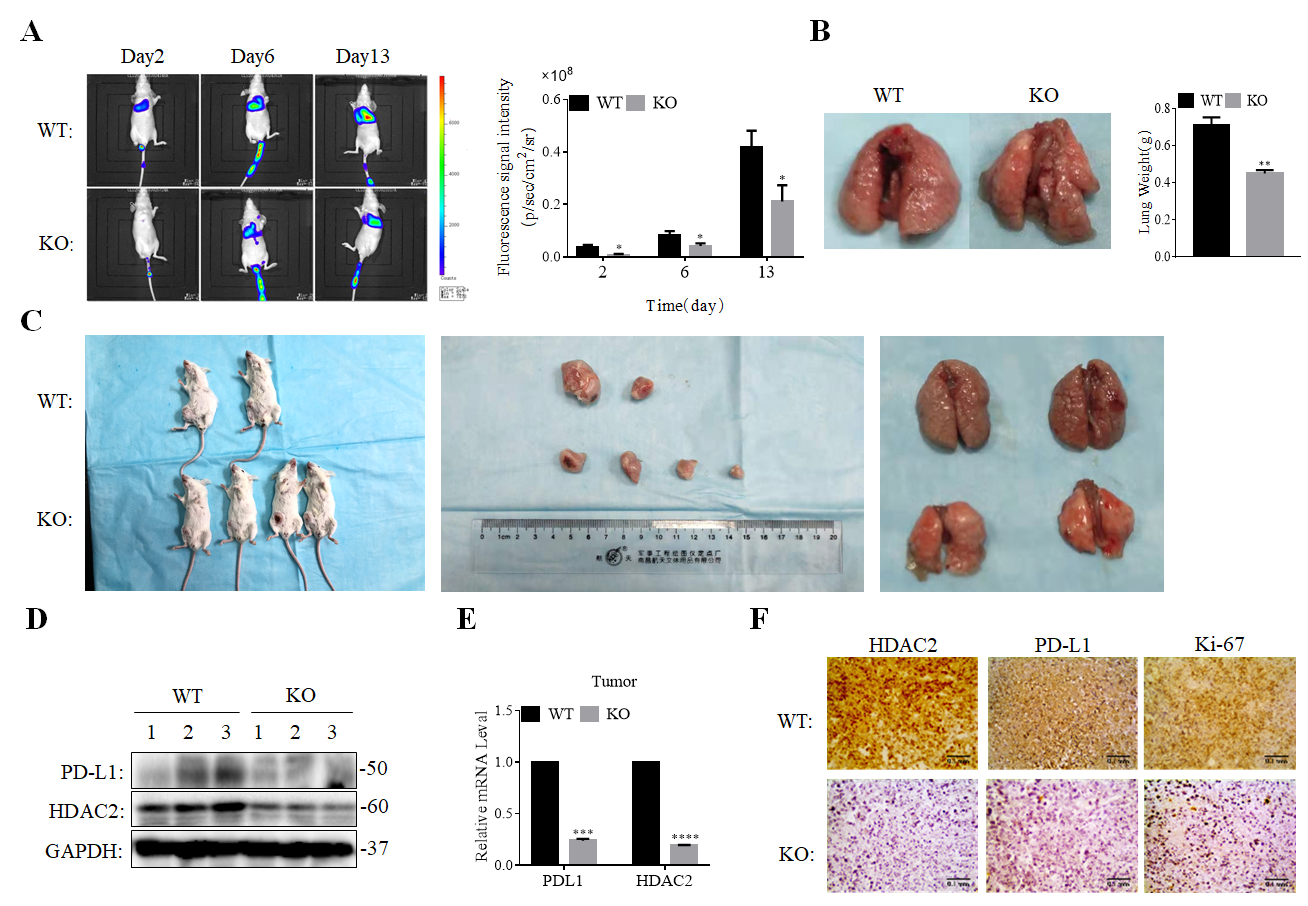

Supplement: Supplementary file 6 — Figure S3 [file 41419_2021_4047_MOESM6_ESM.tif]
